# Statement in Support of: “Virology under the Microscope—a Call for Rational Discourse”

**DOI:** 10.1128/jvi.00451-23

**Published:** 2023-04-25

**Authors:** Peter Speck, Jason Mackenzie, Rowena A. Bull, Barry Slobedman, Heidi Drummer, Johanna Fraser, Lara Herrero, Karla Helbig, Sarah Londrigan, Gregory Moseley, Natalie Prow, Grant Hansman, Robert Edwards, Chantelle Ahlenstiel, Allison Abendroth, David Tscharke, Jody Hobson-Peters, Robson Kriiger-Loterio, Rhys Parry, Glenn Marsh, Emma Harding, David A. Jacques, Matthew J. Gartner, Wen Shi Lee, Julie McAuley, Paola Vaz, Frank Sainsbury, Michelle D. Tate, Jane Sinclair, Allison Imrie, Stephen Rawlinson, Andrew Harman, Jillian M. Carr, Ebony A. Monson, Merilyn Hibma, Timothy J. Mahony, Thomas Tu, Robert J. Center, Lok Bahadur Shrestha, Robyn Hall, Morgyn Warner, Vernon Ward, Danielle E. Anderson, Nicholas S. Eyre, Natalie E. Netzler, Alison J. Peel, Peter Revill, Michael Beard, Alistair R. Legione, Alexandra J. Spencer, Adi Idris, Jade Forwood, Subir Sarker, Damian F. J. Purcell, Nathan Bartlett, Joshua M. Deerain, Bruce J. Brew, Sassan Asgari, Helen Farrell, Alexander Khromykh, Daniel Enosi Tuipulotu, David Anderson, Sevim Mese, Yaman Tayyar, Kathryn Edenborough, Jasim Muhammad Uddin, Abrar Hussain, Connor J. I. Daymond, Jacinta Agius, Karyn N. Johnson, Paniz Shirmast, Mahdi Abedinzadeshahri, Robin MacDiarmid, Caroline L. Ashley, Jay Laws, Lucy L. Furfaro, Thomas D. Burton, Stephen M. R. Johnson, Zahra Telikani, Mary Petrone, Justin A. Roby, Carolyn Samer, Andreas Suhrbier, April Van Der Kamp, Anthony Cunningham, Celeste Donato, Jackie Mahar, Wesley D. Black, Subhash Vasudevan, Roman Lenchine, Kirsten Spann, Daniel J. Rawle, Penny Rudd, Jessica Neil, Richard Kingston, Timothy P. Newsome, Ki Wook Kim, Johnson Mak, Kym Lowry, Nathan Bryant, Joanne Meers, Jason A. Roberts, Nigel McMillan, Larisa I. Labzin, Andrii Slonchak, Leon E. Hugo, Bennett Henzeler, Natalee D. Newton, Cassandra T. David, Patrick C. Reading, Camille Esneau, Tatiana Briody, Najla Nasr, Donna McNeale, Brian McSharry, Omid Fakhri, Bethany A. Horsburgh, Grant Logan, Paul Howley, Paul Young

**Affiliations:** a Flinders University, Bedford Park, South Australia; b Department of Microbiology and Immunology, The University of Melbourne at the Peter Doherty Institute, Melbourne, Victoria, Australia; c Kirby Institute, University of New South Wales, Sydney, Australia; d The University of Sydney, New South Wales, Australia; e Burnet Institute, Melbourne, Victoria, Australia; f Monash University, Melbourne, Victoria, Australia; g Griffith University, Southport, Queensland, Australia; h La Trobe University, Melbourne, Victoria, Australia; i Hull York Medical School, University of York, York, United Kingdom; j Australian National University, Canberra, Australian Capital Territory, Australia; k University of Queensland, St. Lucia, Queensland, Australia; l Commonwealth Scientific and Industrial Research Organisation, Geelong, Victoria, Australia; m University of New South Wales, Sydney, New South Wales, Australia; n University of Melbourne, Melbourne, Victoria, Australia; o Griffith University, Nathan, Queensland, Australia; p Hudson Institute of Medical Research, Clayton, Victoria, Australia; q University of Western Australia, Perth, Western Australia, Australia; r Westmead Institute for Medical Research, Westmead, New South Wales, Australia; s University of Otago, Dunedin, New Zealand; t Ausvet Pty Ltd., Canberra, Australian Capital Territory, Australia; u Commonwealth Scientific and Industrial Research Organisation, Black Mountain, Australian Capital Territory, Australia; v University of Adelaide, Adelaide, South Australia, Australia; w SA Pathology, Adelaide, South Australia, Australia; x University of Otago, Dunedin, New Zealand; y University of Auckland, Auckland, New Zealand; z Maurice Wilkins Centre of Research Excellence, Auckland, New Zealand; aa Victorian Infectious Diseases Reference Laboratory, Royal Melbourne Hospital at the Peter Doherty Institute for Infection and Immunity, Melbourne, Victoria, Australia; bb The University of Newcastle, Newcastle, New South Wales, Australia; cc Queensland University of Technology, Brisbane, Queensland, Australia; dd Charles Sturt University, Wagga Wagga, New South Wales, Australia; ee University of Notre Dame, Sydney, New South Wales, Australia; ff St. Vincent’s Hospital, Sydney, New South Wales, Australia; gg Istanbul University, Istanbul, Turkey; hh Prorenata Biotech, Moledinar, Queensland, Australia; ii Murdoch University, Murdoch, Western Australia, Australia; jj Balochistan University of Information Technology, Engineering and Management Sciences, Quetta, Pakistan; kk QIMR Berghofer Medical Research Institute, Herston, Queensland, Australia; ll Murdoch Children’s Research Institute, Melbourne, Victoria, Australia; mm Biotopia Environmental Assessment Pty Ltd., Melbourne, Victoria, Australia; nn Duke-NUS Medical School, Singapore; oo Islamic Azad University, Mashhad, Iran; pp The New Zealand Institute for Plant & Food Research Limited, Auckland, New Zealand; qq WHO Collaborating Centre for Reference and Research on Influenza, Melbourne, Australia; rr Children’s Medical Research Institute, Westmead, NSW, Australia; ss Vaxmed Pty Ltd., Berwick, Victoria, Australia

**Keywords:** COVID-19, SARS-CoV2, biosafety, coronavirus, gain of function, pandemic, zoonosis

## LETTER

We, members of the Australasian Virology Society, agree with and support the statement entitled “Virology under the Microscope—a Call for Rational Discourse” (1). Like virologists everywhere, we have worked with scientist and clinician colleagues worldwide to develop knowledge, tests, and interventions which collectively have reduced the number of deaths due to COVID-19 and curtailed its economic impact. Such work adds to the extraordinary achievements resulting from virology research that have delivered vaccines and/or antivirals against a long list of diseases and global scourges, including AIDS, smallpox, and polio (1).

We believe the question of the origin of SARS-CoV-2 should be approached with an open mind and in consideration of the best scientific evidence available. We concur with the view that the zoonosis hypothesis has the strongest supporting evidence (2–4), and this is a scenario that has been observed repeatedly in the past (5), including in Australia (6). Recent data strongly support the zoonosis hypothesis (7). We share the concern that emotive and fear-based dialogues in this area add to public confusion and can lead to ill-informed condemnation of virology research.

We believe the current narrative used by some parties—that gain-of-function research is synonymous with high-risk or nefarious activity—fails to appreciate, first, the true scientific value of this legitimate approach to experimental design and, second, the strength and effectiveness of current regulations. There is an extensive history of gain-of-function research safely and effectively contributing to the development of vaccines and antivirals (1). A recent review of gain-of-function studies conducted by the Australian Government defined gain-of-function research as “a change to the genome of any biological entity—a living organism such as an animal, insect, plant, virus, bacterium, or fungus—through any process so that it acquires a new or enhanced function”. The review concluded that oversight of gain-of-function research in Australia is comprehensive and robust (8).

We do not believe virology research needs additional legislative controls. As in the United States, regulations in our region applying to virology research are strong, effective, and provide powerful oversight of manipulations of viruses by researchers. We support the call to legislators to resist fear-based campaigns that might lead to unnecessary and counter-productive restrictions being placed on virology research and may limit progress toward new antiviral drugs and vaccines.

We echo the call for policy makers, virologists, and biosafety experts to work together to ensure that research is conducted safely, with the common goal of reducing the burden of disease caused by viruses.
